# Predicting Responses to Psychedelics: A Prospective Study

**DOI:** 10.3389/fphar.2018.00897

**Published:** 2018-11-02

**Authors:** Eline C. H. M. Haijen, Mendel Kaelen, Leor Roseman, Christopher Timmermann, Hannes Kettner, Suzanne Russ, David Nutt, Richard E. Daws, Adam D. G. Hampshire, Romy Lorenz, Robin L. Carhart-Harris

**Affiliations:** ^1^Psychedelic Research Group, Neuropsychopharmacology Unit, Centre for Psychiatry, Division of Brain Sciences, Department of Medicine, Imperial College London, London, United Kingdom; ^2^The Computational, Cognitive and Clinical Neuroimaging Laboratory (C^*3*^NL), Department of Medicine, Imperial College London, London, United Kingdom; ^3^Psychology Program, Department of Social Sciences, Dickinson State University, Dickinson, ND, United States

**Keywords:** psychedelics, predicting response, well-being, acute effects, peak experience, mystical experience, challenging experience, set and setting

## Abstract

Responses to psychedelics are notoriously difficult to predict, yet significant work is currently underway to assess their therapeutic potential and the level of interest in psychedelics among the general public appears to be increasing. We aimed to collect prospective data in order to improve our ability to predict acute- and longer-term responses to psychedelics. Individuals who planned to take a psychedelic through their own initiative participated in an online survey (www.psychedelicsurvey.com). Traits and variables relating to set, setting and the acute psychedelic experience were measured at five different time points before and after the experience. Principle component and regression methods were used to analyse the data. Sample sizes for the five time points were *N* = 654, *N* = 535, *N* = 379, *N* = 315, and *N* = 212 respectively. Psychological well-being was increased 2 weeks after a psychedelic experience and remained at this level after 4 weeks. Higher ratings of a “mystical-type experience” had a positive effect on the change in well-being after a psychedelic experience, whereas the other acute psychedelic experience measures, i.e., “challenging experience” and “visual effects”, did not influence the change in well-being after the psychedelic experience. Having “clear intentions” for the experience was conducive to mystical-type experiences. Having a positive “set” as well as having the experience with intentions related to “recreation” were both found to decrease the likelihood of having a challenging experience. The baseline trait “absorption” and higher drug doses promoted all aspects of the acute experience, i.e., mystical-type and challenging experiences, as well as visual effects. When comparing the relative contribution of different types of variables in explaining the variance in the change in well-being, it seemed that baseline trait variables had the strongest effect on the change in well-being after a psychedelic experience. These results confirm the importance of extra-pharmacological factors in determining responses to a psychedelic. We view this study as an early step towards the development of empirical guidelines that can evolve and improve iteratively with the ultimate purpose of guiding crucial clinical decisions about whether, when, where and how to dose with a psychedelic, thus helping to mitigate risks while maximizing potential benefits in an evidence-based manner.

## Introduction

There has been notable increase in the volume of clinical research on serotonergic psychedelics within the last decade (Carhart-Harris and Goodwin, 2017). Naturally occurring psychedelics such as psilocybin-containing mushrooms have been used as medicines for centuries, and in the mid twentieth century, some of these and the newly synthesized psychedelic LSD, were briefly explored by the Western medicine. The present revival of interest in psychedelics has been described as a “renaissance” (Sessa, 2012). One particularly notable finding within the present era is that a single dose of psilocybin led to an increase of psychological well-being that endured for at least one year (Griffiths et al., 2008, 2011; Garcia-Romeu et al., 2014). Recent results have suggested that diverse clinical populations can benefit from psychedelics, with rapid and enduring improvements in mental health outcomes seen after treatment with psychedelics for a range of different disorders (Griffiths and Grob, 2010; Anderson et al., 2012; Bogenschutz and Pommy, 2012; Bogenschutz and Johnson, 2016; Mithoefer et al., 2016; Johnson and Griffiths, 2017; Nichols et al., 2017), for a review see Carhart-Harris and Goodwin (2017). Despite these developments, little progress has been made in our ability to predict, ahead of time, the nature of individual responses to a psychedelic (although see Carrillo et al., 2018).

The importance of assessing the potential of psychedelics to treat major psychiatric disorders such as depression and addiction, that are highly prevalent, costly, and for which current treatments have significant limitations (Chisholm et al., 2016), is becoming increasingly well recognized (Carhart-Harris and Goodwin, 2017; Carhart-Harris et al., 2017). However, given the improvements in well-being seen in healthy volunteers after experiences with psychedelics (Griffiths et al., 2006, 2008), it also seems relevant to consider their effects in the general population, not least because prevalence of use in the West remains high (Krebs and Johansen, 2013a,b; Winstock, 2017) and will increase considerably if current efforts to medicalise psychedelics are successful.

A limited number of previous studies have attempted to assess factors that may be predictive of acute or longer-term responses to psychedelics. Perhaps the earliest was carried out by Leary et al. (1963) who found that the level of reported apprehension before taking psilocybin was negatively correlated with the pleasantness of the subsequent experience and willingness to repeat it (Leary et al., 1963). A related finding of anticipatory anxiety predicting acute anxiety during a psychedelic experience was reported by the same team (Metzner et al., 1965). Richards et al. (1977) found that those who had a “peak experience” (Maslow, 1959, 1964, 1968), described as experiencing disorientation in space and time, feelings of being free of inner conflict, feelings of awe, amazement and humility, and a sense of oneness with the universe, were less hostile, tense and anxious beforehand. The authors explained this by the observation that these individuals were more engaged in the therapeutic process, more willing to confront anxiety and less frightened by the prospect of self-confrontation (Richards et al., 1977). Similarly, Metzner et al. (1965) found that those with positive prior expectations for their experience did indeed have more positive experiences under a psychedelic (Metzner et al., 1965).

The most notable modern study to have assessed prediction of response was carried out by Studerus et al. (2012). Here, data pooled from 23 controlled studies involving over 250 participants was used to identify factors predictive of response to psilocybin. The findings included that baseline trait “absorption” and being psychologically “well” in the days prior to the experience, were predictive of peak experiences, whereas younger age, emotional excitability prior to the experience, and being in a positron emission tomography scanner, were all predictive of unpleasant, “challenging experiences” (i.e., experiences characterized by anxiety, psychological struggle, fear, panic and/or paranoia).

While this study is useful, it is limited to predicting just the acute experience with psilocybin and does not address longer-term effects or responses to other psychedelics. Neither is it reflective of the importance of non-pharmacological variables present when psychedelics are used therapeutically. Regarding longer-term outcomes, a growing number of controlled studies with psychedelics are endorsing the view that the occurrence of a “mystical-type” experience is predictive of positive long-term outcomes (O'Reilly and Funk, 1964; Klavetter and Mogar, 1967; Pahnke et al., 1970; Kurland et al., 1972; Richards et al., 1977; Griffiths et al., 2011, 2016; MacLean et al., 2011; Garcia-Romeu et al., 2014; Bogenschutz et al., 2015; Ross et al., 2016; Roseman et al., 2017). The term “mystical-type experience” is based on Walter Stace's work on mysticism (Stace, 1960), and is effectively synonymous with the term peak experience, derived from the work of psychologist Abraham Maslow (Maslow, 1959, 1964) described earlier. There is mixed data on the relationship between challenging psychological experiences and longer-term outcomes, with one study suggesting that challenging experiences can be beneficial, but not if the duration of struggle dominates the entire experience (Carbonaro et al., 2016). It has also been shown that the personality trait neuroticism is predictive of the occurrence of a challenging experience (Barrett et al., 2017).

The present study was conceived as a way of reducing uncertainty about how an individual might respond to a psychedelic. Our hope is that such an endeavor may ultimately serve to guide critical decisions about whether, when and how to dose for a psychedelic experience. We wished to combine predictions of acute experience with those of longer-term changes, to track, within the same time-limited study, the processes of potential change occurring within individuals after a psychedelic experience. To do this, we set up a web-based survey embedded within a purpose-built website^1^ that could record subjective data at five crucial time points: (1) 1 week before, (2) within 1 day before, (3) within a few days after, (4) 2 weeks after, and (5) 4 weeks after a psychedelic experience.

Individuals were invited to sign up for the survey if they planned to have a psychedelic experience within the near future. After electronically declaring informed consent, participants stated the expected date for their experience and provided their email addresses via which reminders were sent for them to complete the relevant surveys at the relevant time points. Measures were used to assess psychological well-being (which served as the primary outcome) as well as the acute psychedelic experience. Data reduction methods were used to generate principal components underlying “set” and “setting” factors as well as a variety of intentions for the psychedelic experience, which served as independent variables. A range of potential trait predictor variables were measured at baseline and are listed in the methods section.

Our primary hypothesis was that subjective well-being would be significantly increased 2 weeks after the psychedelic experience compared to baseline. Secondly, it was hypothesized that the nature of the acute psychedelic experience would be predictive of subsequent changes in well-being (Roseman et al., 2017). Specifically, it was hypothesized that mystical-type experiences (Stace, 1960) under the psychedelic would be positively related to subsequent improvements in well-being. The contribution of “set” factors relating to the pre-state (i.e., one's state of mind immediately prior to taking the psychedelic), setting, as well as different trait variables and intentions for having the psychedelic experience to the subsequent nature of the acute psychedelic experience were assessed.

## Materials and methods

### Study design

**Figure 1:**
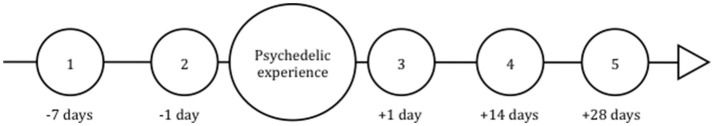
Study timeline. The numbers presented represent the five time points of measurement.

This was a prospective study design using opportunity sampling and web-based data collection. The inclusion criteria were: at least 18 years old, good comprehension of the English language and having the intention to take a classic psychedelic drug (psilocybin/magic mushrooms/truffles, LSD/1P-LSD, ayahuasca, DMT/5-MeO-DMT, salivia divinorum, mescaline, or iboga/ibogaine) in the near future. This approach provided the opportunity to collect a large amount of data in a non-controlled, naturalistic and observational manner. The study consisted of a total of five surveys completed at different moments. The first survey was completed one week before the planned psychedelic experience. One day before the experience, the second survey was completed, followed by the third survey one day after the psychedelic experience. The fourth and fifth surveys were completed at two and four weeks after the psychedelic experience, respectively (see Figure 1).

### Participant recruitment and dissemination of the study

A website^2^ was created in collaboration with a team of web designers. This website contained all information needed for individuals to be informed about the study design, what was expected from participants and the informed consent. Once the informed consent was read and agreed on, individuals were able to sign up on the website by providing their name, email address and the date on which they expected to have their experience. Online advertisements, including a link to the main website that hosted the survey, were posted and shared on Facebook, Twitter, email newsletters, and online drug forums^3^,^4^. Once participants signed up, they were included in an emailing system that was programmed to send out emails and reminders at specific times depending on the anticipated date of the psychedelic experience provided by the participants in the sign up process. Emails contained links to the relevant surveys, which were implemented and hosted by the online service system Survey Gizmo^5^.

### Ethical considerations

The aim of the present study was to sample variables associated with psychedelic drug use, without manipulating or promoting such use. A disclaimer text on the website was included stating: “This survey should not be viewed as advocacy of psychedelic drug use. Its aim is to sample people whose intent to take a psychedelic is already established.”

Participants provided their name and email address in the sign up process, however this information was not saved in the survey responses nor used while handling the data. This data was only used to personalize the emails needed to send out the relevant survey links. When emails were sent out including the survey links, a unique identification code was generated which was included in the survey links as a uniform resource locator (URL). This offered the opportunity to identify and link multiple survey responses of one individual without sampling privacy-sensitive information. Furthermore, Survey Gizmo has features that protect the security of responses, in line with the ethics requirements. The study was approved by Imperial College Research Ethics Committee (ICREC) and the Joint Research Compliance Office (JRCO) at Imperial College London.

### The surveys

All surveys consisted of a large battery of existing measures, and in some cases self-constructed items were included that asked about a particular phenomenon of interest and for which there were no known validated measures available. Only key measures of interest that will be discussed in this paper will be mentioned in the measures section, the remaining measures will be discussed elsewhere in future papers.

### Survey 1: baseline

#### Structure

The first survey asked about demographic information such as age, sex, nationality, native language, educational background, employment status, history of psychiatric illness, and previous drug use. After providing this information, participants were asked to indicate their relationship to psychedelics according to a set of statements. This served to assess potential sample bias.

Next, participants stated the specific psychedelic they expected to take: psilocybin/magic mushrooms/truffles, LSD/1P-LSD, ayahuasca, DMT/5-MeO-DMT, salvia divinorum, mescaline, iboga/ibogaine, or the option to give a free answer. Subsequently, participants were asked what their motives or intentions were for the experience from the following: fun/recreational/party, therapeutic/personal growth, medicinal, spiritual experience, religious experience, curiosity, social, connection with nature, to escape from difficult emotions, and to confront difficult emotions. Each motive was rated as “not at all,” “somewhat,” “moderately,” or “very much.” The remaining pages of the first survey contained baseline measures assessing trait variables we felt may be predictive of response.

#### Measures

The Warwick-Edinburgh Mental Wellbeing Scale (WEMWBS) (Tennant et al., 2007) was used to assess psychological well-being. This questionnaire contains 14 items and covers concepts associated with positive mental health, such as positive affect, satisfying interpersonal relationships, positive functioning, and hedonic and eudaimonic aspects. The Ten-Item Personality Inventory (TIPI) (Gosling et al., 2003) was included as a measure for personality. This questionnaire contains 10 items covering the five major personality domains identified by Costa and MacCrae (1992): openness to experience, extraversion, agreeableness, conscientiousness, and emotional stability (the inverse of neuroticism), and has been shown to be a valid substitute for the longer “Big-Five” instruments (Costa and MacCrae, 1992). The modified version of the Tellegen Absorption Scale (MODTAS) (Tellegen and Atkinson, 1974) was used to measure trait absorption. For brevity, only the 25 scored items were included in this survey. Further, the short version of the Spielberger State-Trait Anxiety Inventory (STAI-SF) (Marteau and Bekker, 1992) was used to measure trait anxiety. The complete form of the widely used STAI contains 20 items, whereas the validated short version consists of six items. The Short Suggestibility Scale (SSS) (Kotov et al., 2004) is a shorter version of the full Multidimensional Iowa Suggestibility Scale (MISS) consisting of various subscales. The SSS contains 21 items originating from the full scale's subscales: consumer and physiological suggestibility, persuadability, peer conformity, and physiological reactivity. The Stubborn Opinionatedness scale (SOP) (Kotov et al., 2004) was developed as a companion construct to the suggestibility scale, covering stubbornness, or unpersuadability, to measure how stubborn and opinionated an individual is. This questionnaire contains 16 items.

#### Duration

The completion time of the first survey was about 35 min.

#### Timing

This survey was sent to the participants exactly 1 week before they expected to have their psychedelic experience, or immediately after they signed up, if the date of their psychedelic experience was within the same week as this.

### Survey 2: pre-state

#### Structure

The items of the second survey aimed to capture thoughts and expectations right before the experience and the willingness to open up- and surrender to the experience, or “set,” which will be discussed in the measures section below.

#### Measures

To our knowledge, until now, no validated and published questionnaire existed that measures an individual's state of mind- “set” just before drug intake. For this reason, we constructed a measure containing items aimed at capturing this (see Table 3). These items were inspired by the work of Suzanne Russ and her working on meditation (Russ and Elliott, 2017) and psychedelics (Russ et al., unpublished).

#### Duration

It took approximately 5 min to complete the second survey.

#### Timing

This survey was sent to the participants 1 day before they expected to have their psychedelic experience, or immediately after they signed up, if the date of their psychedelic experience was the day after they signed up.

### Survey 3: reporting on the acute psychedelic experience

#### Structure

Participants were asked what specific psychedelic they took. Options included: psilocybin/magic mushrooms/truffles, LSD/1P-LSD, ayahuasca, DMT/5-MeO-DMT, salvia divinorum, mescaline, iboga/ibogaine, or the option to give a free answer. Next, it was asked what dose was used, which was assessed using a previously used approach of estimating in LSD equivalents, so to provide a reference-standard (Nour et al., 2016), options were: a low dose (no more than 50 micrograms of LSD), a moderate dose (no more than 100 micrograms of LSD), a high dose (no more than 200 micrograms of LSD), a very high dose (no more than 300 micrograms of LSD), or an extremely high dose (more than 300 micrograms of LSD). The remaining pages of the third survey contained measures assessing acute effects of the psychedelic experience. The relevant measures for this paper are listed below.

#### Measures

The Altered States of Consciousness rating scale (OAV or 11D-ASC) (Studerus et al., 2010) is a measure to assess altered states of consciousness, which are transient deviations from an individual's normal waking consciousness. This scale consists of 42 scored items covering 11 subscales (i.e., impaired control and cognition, spiritual experience, blissful state, elementary imagery, unity, audio-visual synaesthesia, disembodiment, changed meaning of percepts, anxiety, complex imagery, and insightfulness) and has been widely used to measure the acute effects of psychedelic drugs. For this study, we created one measure for “visual effects” using the scores of the elementary imagery, complex imagery and audio-visual synaesthesia subscales. The Mystical Experience Questionnaire (MEQ) (Barrett et al., 2015) contains 30 items leading to four subscale scores (i.e., mystical, positive mood, transcendence of time and space, and ineffability) (MacLean et al., 2012) covering aspects relating to mystical experiences, and a total score. The Challenging Experience Questionnaire (CEQ) (Barrett et al., 2016) contains 30 items leading to a total score and seven subscales scores (i.e., fear, grief, physical distress, insanity, isolation, death, and paranoia), covering acute affective, cognitive, and somatic symptoms. This questionnaire was developed to assess unpleasant reactions to psychedelic drugs, or “challenging” experiences. Also, aspects of the setting of the psychedelic experience were assessed in the third survey, using the following items: “Did your experience take place within a psychedelic drug retreat?,” “Was the setting designed and/or prepared with a therapeutic objective in mind?,” and “Was the setting more designed and/or suited for a recreational and/or social occasion, such as a party?,” which were all answered with either yes or no. Next, it was asked to indicate how many people were present during the majority of the experience, options included: “1 (only myself),” “between 2 and 5,” “between 6 and 15,” “between 16 and 30,” “between 31 and 100,” “more than 100.” Also, it was asked whether individuals were present who took responsibility for, or looked after, the participant during the psychedelic experience, which was answered with either yes or no.

#### Duration

The completion time of the third survey was about 30 min.

#### Timing

This survey was sent to the participants 1 day after they were due to have their psychedelic experience.

### Survey 4: 2 weeks afterwards

#### Structure

The fourth survey asked whether or not participants have had more than one psychedelic experience in the past 2 weeks, and if so, how many. The remaining part of the fourth survey mainly contained the same measures as those included in the first survey. The only variable that was of interest here was well-being, because of the interest in the change scores of this construct before and after the psychedelic experience.

#### Duration

The completion time of the fourth survey was about 35 min.

#### Timing

This survey was sent to the participants 2 weeks after they had their psychedelic experience, based on the date they provided in the sign up process.

### Survey 5: 4 weeks afterwards

#### Structure

The fifth survey asked whether or not participants have had more than one psychedelic experience in the past 4 weeks, and if so, how many. The remaining part of the fifth survey mainly contained the same measures as those included in the first and fourth survey. The only variable that was of interest here was well-being, because of the interest in the change scores of this construct before and after the psychedelic experience.

#### Duration

The completion time of the fifth survey was about 35 min.

#### Timing

This survey was sent to the participants 4 weeks after they had their psychedelic experience, based on the date they provided in the sign up process.

### Statistical analyses

A principle component analysis was done to the set and setting items that were self-constructed (see Table 3) and for the 10 different intentions to have a psychedelic experience (see Table 4). This was done to reduce dimensionality and to find a general structure underlying these overlapping items, which could then be used in the subsequent analyses. Both principle component analyses were performed using IBM SPSS Statistics version 24, using the principle component method and an orthogonal rotation (Varimax). The appropriate number of factors was determined after investigating Cattell's scree plot and by the cut-off of eigenvalues larger than one.

A GLM repeated measures ANOVA was performed to evaluate the changes in well-being scores. WEMWBS scores were included as dependent variable with time as within-subject effect. The following variables were included as covariates to control for confounding effects on well-being scores: *age, sex, employment status, educational level, number of lifetime uses of a psychedelic, multiple psychedelic experiences during their enrolment in the study*, and four items asking about the individual's relationship with psychedelic drugs: “*I am an active advocate of psychedelic drug use*,” *I am an active advocate of the therapeutic use of psychedelics*,” *I have an advanced knowledge about psychedelics*,” and “*I am a highly experienced psychedelic drug user*.” Only the covariates that showed a significant effect will be included in the further analyses.

To further investigate the changes in well-being, independent *t*-tests have been performed to compare mean values for the following variables between those who decreased in well-being compared to those who did not show any change, or increased in well-being: baseline *WEMWBS* score, the personality traits *openness to experience (TIPI), emotional stability (TIPI), absorption (MODTAS), suggestibility (SSS), stubbornness (SOP), trait anxiety (STAI)*, the three components underlying intentions: “*spiritual connection*,” “*recreation*” and “*emotional*,” the three components underlying set and setting: “*set*,” “*setting*,” and “*clear intentions*,” *drug dose*, the variable *being in a therapeutic environment*, the total scores of the *MEQ* and *CEQ* and the *visual effects* score, based on the elementary imagery, complex imagery and audio-visual synaesthesia subscales of the OAV.

To examine relationships between variables of interest, linear mixed models were used in the MIXED procedure of IBM SPSS Statistics version 24. A mixed model analysis was chosen because of its ability to handle missing data and use all existing data. The model following from our hypothesis included the WEMWBS as the dependent variable and time as a repeated effect, with an unstructured covariance structure. This model contained in the fixed part: *time, number of lifetime uses of a psychedelic*, the item “*I am a highly experienced psychedelic drug user*”, *MEQ, CEQ, visual effects*, and all two-way interaction terms between time and the variables: *number of lifetime uses of a psychedelic*, the item “*I am a highly experienced psychedelic drug user*”, *MEQ, CEQ*, and *visual effects*.

The next model was included to assess what type of variable (i.e., traits, set and setting, intentions, drug dose, or acute psychedelic measures) was most influential in predicting well-being changes. The model included the WEMWBS as the dependent variable and time as a repeated effect, with an unstructured covariance structure. This model contained in the fixed part: *time, number of lifetime uses of a psychedelic*, the item “*I am a highly experienced psychedelic drug user*,” the personality traits *openness to experience (TIPI), emotional stability (TIPI), absorption (MODTAS), suggestibility (SSS), stubbornness (SOP), trait anxiety (STAI)*, the three components underlying intentions: “*spiritual connection*,” “*recreation*,” and “*emotional*,” the three components underlying set and setting: “*set*,” “*setting*,” and “*clear intentions*,” *drug dose*, the variable *being in a therapeutic environment*, the total scores of the *MEQ* and *CEQ, visual effects*, and all two-way interaction terms between *time* and the variables described above.

Next, a GLM multivariate linear regression model was used to evaluate what variables were able to predict measures related to the acute psychedelic experience. The total scores of the MEQ, CEQ and visual effects were used as dependent variables. Included as independent variables were: *number of lifetime uses of a psychedelic*, the item “*I am a highly experienced psychedelic drug user*,” the personality traits *openness to experience (TIPI), emotional stability (TIPI), absorption (MODTAS), suggestibility (SSS), stubbornness (SOP), trait anxiety (STAI)*, the three components underlying intentions: “*spiritual connection*,” “*recreation*,” and “*emotional*,” the three components underlying set and setting: “*set*,” “*setting*,” and “*clear intentions*,” *drug dose*, and the variable *being in a therapeutic environment*.

Tests for multicollinearity between variables have been done before running the analyses. All variables were standardized before being entered into the analyses. Lastly, for all statistical tests, an alpha of 0.050 was used.

## Results

### Demographics

**Figure 2:**
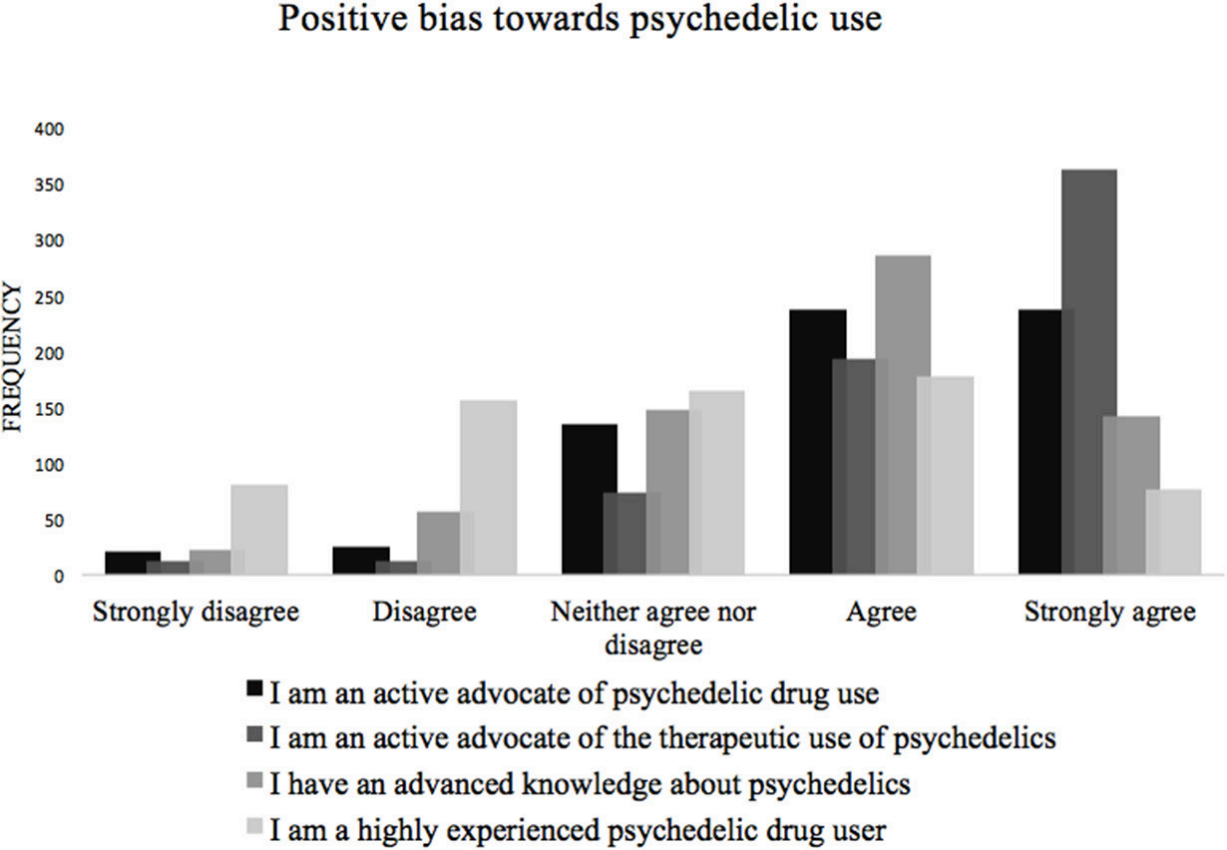
Four items assessing participants' attitude towards psychedelic drugs measured in the first survey.

**Figure 3:**
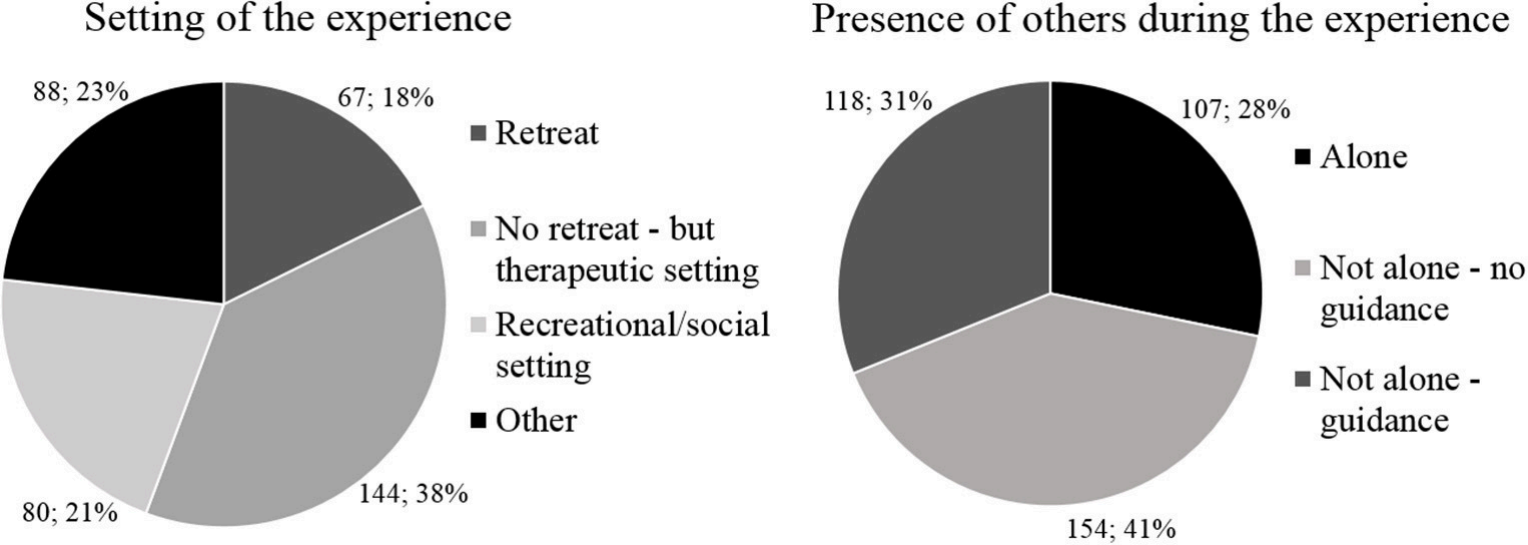
Information regarding the setting of the psychedelic experience collected at the third time-point. Absolute and relative frequencies are shown.

The following sample sizes were collected for each of the five surveys respectively: *N* = 654, *N* = 535, *N* = 379, *N* = 315, and *N* = 212. See Table 1 for demographic information that was collected in the first survey. Figure 2 shows that the study sample was positively biased towards psychedelic drug use in general and even more so for therapeutic use of psychedelics, which was expected. See Table 2 and Figure 3 for demographic information of the psychedelic experience collected in the third survey, 1 day after the psychedelic experience. In the fourth and fifth survey, participants were asked whether they had multiple psychedelic experiences during the time they were enrolled in the study. At the fourth and fifth time-point, 88 and an additional 29 participants respectively, said to have had more than one psychedelic experience than the experience they signed up with.

**Table 1:** Demographic data collected at the first time point.

**Total**		**654**
Gender	Male	485 (74.2%)
	Female	165 (25.2%)
	Other	4 (0.6%)
Age		28.9 ±10.4
Educational level	Left school before age 16 without qualifications	8 (1.2%)
	Some high school/GCSE level (in UK)	45 (6.9%)
	High school diploma/A-level education (in UK)	97 (14.8%)
	Some university (or equivalent)	179 (27.4%)
	Bachelor's degree (or equivalent)	193 (29.5%)
	Post-graduate degree (e.g., Masters or Doctorate)	132 (20.2%)
Employment status	Student	256 (39.1%)
	Unemployed	53 (8.1%)
	Part-time job	98 (15.0%)
	Full-time job	237 (36.2%)
	Retired	10 (1.5%)
Nationality	United States	199 (30.4%)
	United Kingdom	128 (19.6%)
	Denmark	60 (9.2%)
	Germany	32 (4.9%)
	Canada	32 (4.9%)
	Other (50 in total)	203 (31.0%)
Psychiatric history	Ever been diagnosed with at least one psychiatric illness in the past^a^	214 (32.7%)
	Never been diagnosed with a psychiatric illness	381 (67.3%)
Previous psychedelic drug use	Never (psychedelic naïve)	62 (9.5%)
	Once	40 (6.1%)
	2–5 times	148 (22.6%)
	6–20 times	215 (32.9%)
	More than 21 times	189 (28.9%)

*Means ± standard deviations and absolute frequencies are shown. Numbers in parenthesis indicate the percentages corresponding to the absolute frequencies*.

a *At least one of the following psychiatric illnesses: major depressive disorder, bipolar disorder, schizophrenia, anxiety disorder, substance abuse disorder, alcohol dependence, hallucinogen persisting perception disorder, psychotic disorder, attention deficit hyperactivity disorder, obsessive compulsive disorder, and eating disorder*.

**Table 2:** Drug variables collected at the third time point.

**Total**		**379**
Drug type	LSD/1P-LSD	183 (48.3%)
	Psilocybin	109 (28.8%)
	Ayahuasca	42 (11.1%)
	DMT/5-MeO-DMT	12 (3.2%)
	Mescaline (Peyote, San Pedro)	10 (2.6%)
	Other	23 (6.1%)
Drug dose^a^	A low dose	39 (10.3%)
	A moderate dose	144 (38.0%)
	A high dose	135 (35.6%)
	A very high dose	37 (9.8%)
	An extremely high dose	24 (6.3%)

*Absolute frequencies and the percentages corresponding to the absolute frequencies are shown*.

a *Drug dose was reported in LSD equivalents: no more than 50 micrograms of LSD, no more than 100 micrograms of LSD, no more than 200 micrograms of LSD, no more than 300 micrograms of LSD, and more than 300 micrograms of LSD, respectively*.

### Principle component analysis: set and setting

A total of 12 self-constructed items regarding set and setting (see Table 3) were included in the principle component analysis. The Kaiser-Meyer-Olkin measure of sampling adequacy was 0.800 and Bartlett's test of sphericity was highly significant [*X*2(66) = 941.23, *p* < 0.001], indicating that the data was suitable for a principle component analysis (Budaev, 2010). The structure that was found (see Table 3) consisted of three components, which explained 54.33% variance cumulatively. Based on a study of the loadings, we named these components: (1) “*set,”* (2) “*setting,”* and (3) “c*lear intentions.”* In the same order, the variance explained by each factor was respectively: 31.12, 12.49, and 10.72%.

**Table 3:** Item loadings from the principle component analysis of item scores relating to set and setting.

	**Factor 1: “Set”**	**Factor 2: “Setting”**	**Factor 3: “Clear intentions”**
I feel comfortable about the upcoming experience.	**0.765**	0.067	0.146
I feel open to the upcoming experience.	**0.708**	0.217	0.197
I feel well prepared for the upcoming experience.	**0.648**	0.148	0.336
I feel anxious.	**−0.635**	0.083	0.356
I am in a good mood.	**0.619**	0.242	−0.021
I feel ready to surrender to whatever will be.	**0.583**	0.284	0.088
I am preoccupied with my work and/or life duties.	**−0.392**	−0.102	0.337
I have a good feeling about my relationship with the group/people who will be with me during my experience.	0.181	**0.863**	−0.067
I have a good relationship with the main person/people who will look after me during the upcoming experience.	0.071	**0.848**	−0.105
The environment/setting feels good for my upcoming experience.	0.350	**0.604**	0.243
I have strong expectations for the upcoming experience.	0.023	−0.047	**0.695**
I have a clear intention for the upcoming experience.	0.215	0.048	**0.694**

*The included items were self-constructed and were answered using a visual analog scale with 0 = strongly disagree and 100 = strongly agree. The first 10 items were answered by 535 participants, item 11 was answered by 386 participants, and item 12 was answered by 326 participants. The highest loading of each item is presented in bold*.

### Principle component analysis: intentions

An item was constructed asking about the individual's intentions to have a psychedelic experience (i.e., “Can you indicate what your motives are to undergo a psychedelic session/ceremony/experience?”), followed by a list of 10 motives: (1) fun/recreational/party, (2) therapeutic/personal growth, (3) medicinal, (4) spiritual experience, (5) religious experience, (6) curiosity, (7) social, (8) connection with nature, (9) to escape from difficult emotions, and (10) to confront difficult emotions. Each motive was rated on a four-point Likert scale with the options: “not at all,” “somewhat,” “moderately,” and “very much.” Ratings of these 10 motives were included in the principle component analysis. The Kaiser-Meyer-Olkin measure of sampling adequacy was 0.695 and Bartlett's test of sphericity was highly significant [*X*2(45) = 1130.52, *p* < 0.001], indicating that the data was suitable for a principle component analysis. The structure that was found (see Table 4) consisted of three components, which explained 55.14% variance cumulatively. Based on a study of the loadings, we named these components: (1) “*spiritual connection*,” (2) “*recreation*,” and (3) “*emotional*.” In the same order, the variance explained by each factor was respectively: 26.88, 16.24, and 12.01%. It has to be noted that some of the intentions loaded highly on more than one component. Only the highest loading of each intention was represented in bold below the components to create a clear overview of which intention contributed the most to each component. This does not mean that these intentions were only important for only that component; they still explain variance in other components.

**Table 4:** Item loadings from the principle component analysis of the 10 different intentions to have a psychedelic experience.

	**Factor 1: “Spiritual connection”**	**Factor 2: “Recreation”**	**Factor 3: “Emotional”**
Spiritual experience	**0.779**	−0.073	0.101
Religious experience	**0.664**	0.005	0.045
Connection with nature	**0.636**	0.415	−0.056
Therapeutic/personal growth	**0.511**	−0.296	0.427
Fun/recreational/party	−0.228	**0.763**	−0.231
Social	0.045	**0.753**	−0.088
Curiosity	0.073	**0.432**	0.224
To escape from difficult emotions	−0.238	0.293	**0.760**
To confront difficult emotions	0.278	−0.194	**0.713**
Medicinal	0.466	−0.184	**0.486**

*The included items were self-constructed and were answered using a four-point Likert scale with the options: “not at all,” “somewhat,” “moderately,” and “very much.” The highest loading of each item is presented in bold*.

### Changes in well-being scores

**Figure 4:**
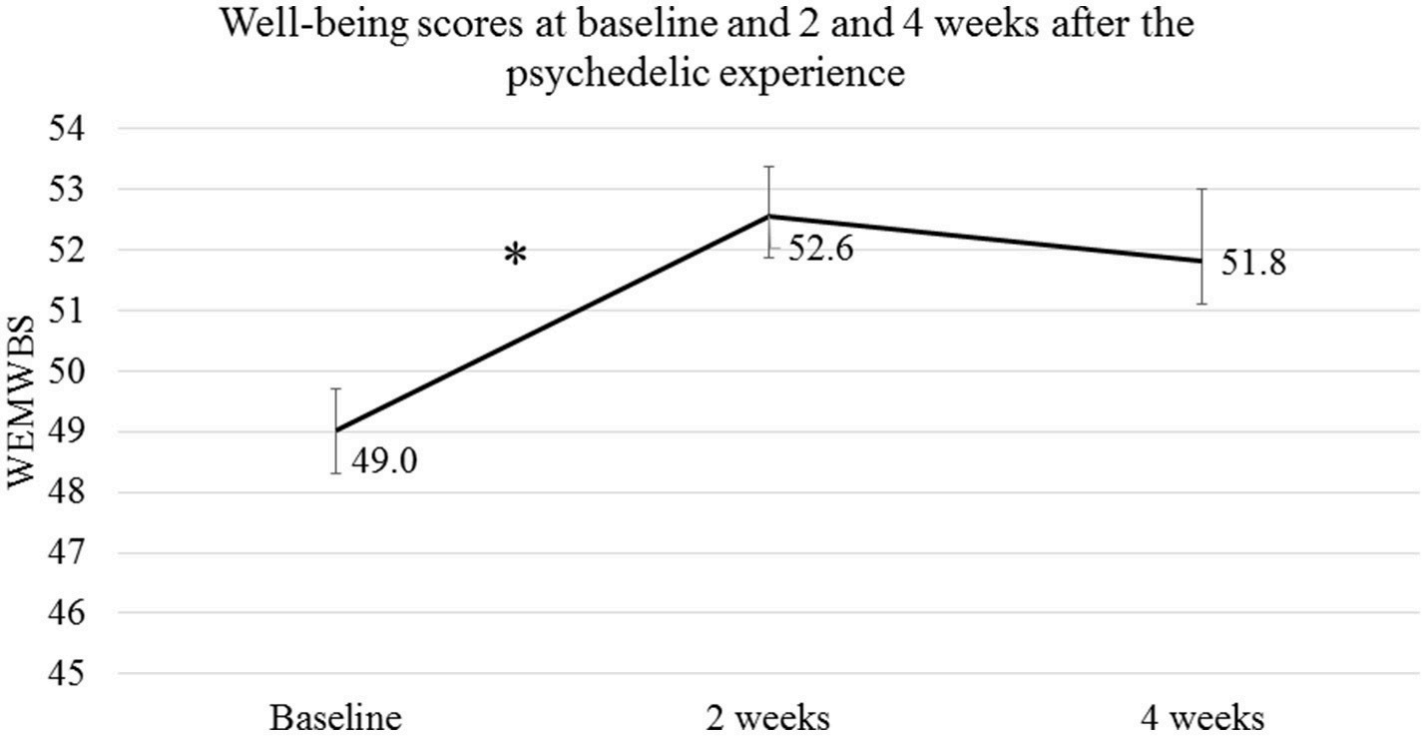
Well-being scores collected at baseline, i.e., 1 week before the psychedelic experience, and at 2 and 4 weeks after the psychedelic experience. Error bars represent the 95% confidence interval. *p < 0.05.

A significant quadratic trend was identified in the WEMWBS scores [*F*_(154)_ = 23.20, *p* < 0.001], showing that well-being scores increased 2 weeks after the psychedelic experience compared to baseline, and remained at this level 4 weeks after the psychedelic experience (see Figure 4). Normative data based on the general population in England and Scotland^6^ showed a mean WEMWBS score of 50.7 (95% CI: 50.3 – 51.1). The current study sample showed a mean well-being score at baseline of 49.0 (95% CI: 48.3 – 49.7), which was below the mean of the normative data, but showed an increased mean WEMWBS score of 52.6 (95% CI: 51.8 – 53.4) at 2 weeks and 51.8 (95% CI: 50.6 – 53.0) at 4 weeks after the psychedelic experience.

### Splitting participants based on change in well-being

Splitting the data file into two subsamples: one in which well-being scores decreased two weeks after the psychedelic experience and another in which there was either no change or an increase, led to sample sizes of *N* = 85 and *N* = 194, respectively. The mean scores of mystical-type and challenging experiences did not differ between the two groups. The only variable that differed between the two subsamples was the mean WEMWBS score at baseline, which was significantly lower in the subsample showing increases in well-being after a psychedelic experience [*F*_(277)_ = 6.95, *p* < 0.001]. This could indicate that participants having lower baseline well-being scores have more scope for positive change in well-being after a psychedelic experience.

### Acute psychedelic experience measures predicting well-being changes

A significant interaction between *time* and the *MEQ* was found on WEMWBS scores [*F*_(232)_ = 3.03, *p* < 0.050], showing that mystical-type experiences positively influenced changes in well-being after the psychedelic experience. Higher ratings of a mystical-type experience had a positive effect on well-being scores measured at two [*t*_(286)_ = 4.52, *p* < 0.001] and four weeks [*t*_(231)_ = 2.46, *p* < 0.020] after a psychedelic experience. No significant interaction effect with time was found for the *CEQ*. However, the *CEQ* was overall negatively associated with WEMWBS scores at baseline [*t*_(275)_ = −3.04, *p* < 0.004] and at 2 weeks [*t*_(275)_ = −4.74, *p* < 0.001] and 4 weeks after the psychedelic experience [*t*_(227)_ = −2.31, *p* < 0.030], but not with the change in well-being scores from baseline. Also, no interaction between time and *visual effects* was found. Further, the *number of lifetime uses of a psychedelic* was negatively associated with WEMWBS scores measured at 2 weeks after the psychedelic experience [*t*_(287)_ = −2.59, *p* < 0.020], meaning that having used psychedelic drugs more often in the past had a negative effect on in well-being measured 2 weeks after the psychedelic experience, which also means that having used psychedelics less often, or being completely psychedelic naïve, had a positive effect on well-being measured 2 weeks after the experience. Lastly, the item “*I am a highly experienced psychedelic drug user*” was significantly associated with WEMWBS scores at baseline [*t*_(345)_ = 4.22, *p* < 0.001] and at two [*t*_(290)_ = 3.75, *p* < 0.001] and 4 weeks [*t*_(230)_ = 3.83, *p* < 0.003] after the psychedelic experience, indicating that individuals who rated themselves as highly experienced with psychedelics had higher well-being scores in general. See Table 5 for the coefficients of the variables included in this model.

**Table 5:** Acute psychedelic experience measures predicting well-being at different time-points.

**Parameter**	**B**	**Std. Error**	***t*-value**	***p*-value**
Intercept	49.716	0.432	115.158	0.000
Time = 4	3.178	0.430	7.390	0.000
Time = 5	2.830	0.486	5.819	0.000
Number of lifetime uses of a psychedelic * Time = 0	−0.030	0.571	−0.052	0.958
Number of lifetime uses of a psychedelic * Time = 4	−1.378	0.532	−2.589	0.010
Number of lifetime uses of a psychedelic * Time = 5	−1.284	0.685	−1.875	0.062
Bias item^a^ * Time = 0	2.511	0.596	4.216	0.000
Bias item^a^ * Time = 4	2.112	0.564	3.745	0.000
Bias item^a^ * Time = 5	2.744	0.716	3.832	0.000
Mystical-type experience * Time = 0	0.948	0.545	1.738	0.083
Mystical-type experience * Time = 4	2.287	0.506	4.516	0.000
Mystical-type experience * Time = 5	1.608	0.653	2.462	0.015
Challenging experience * Time = 0	−1.435	0.473	−3.037	0.003
Challenging experience * Time = 4	−1.975	0.417	−4.735	0.000
Challenging experience * Time = 5	−1.252	0.541	−2.312	0.022
Visual effects * Time = 0	0.595	0.549	1.085	0.279
Visual effects * Time = 4	0.079	0.507	0.156	0.876
Visual effects * Time = 5	0.211	0.670	0.315	0.753

*Dependent variable: WEMWBS score. Effect of each variable on each time-point is given. Time = 0: baseline, Time = 4: two weeks after the psychedelic experience, Time = 5: 4 weeks after the psychedelic experience*.

a Bias item: “I am a highly experienced psychedelic drug user.”

### Traits, set and setting, intentions and acute psychedelic experience measures predicting well-being changes

When entering all variables of interest into one model, it seemed that only baseline *trait anxiety* had an effect on the change in well-being, indicated by the significant interaction between *time* and *trait anxiety* (*F*_(172)_ = 3.93, *p* < 0.030), meaning that baseline *trait anxiety* influenced WEMWBS scores differently at each time-point. *Trait anxiety* was negatively associated with baseline WEMWBS scores [*t*_(178)_ = −5.64, *p* < 0.001] as well as well-being 4 weeks after the psychedelic experience [*t*_(286)_ = −2.32, *p* < 0.030], but not at 2 weeks after the psychedelic experience. There seemed to be a general negative association between baseline *trait anxiety* and well-being, meaning that high baseline levels of *trait anxiety* were associated with low levels of well-being, however this negative association was not significant 2 weeks after the psychedelic experience. Also, this analysis showed a significant interaction between *time* and the *number of lifetime uses of a psychedelic* [*F*_(106)_ = 6.11, *p* < 0.004], meaning that the *number of lifetime uses of a psychedelic* influenced the WEMWBS scores differently at each time-point. The *number of lifetime uses of a psychedelic* did not relate to baseline WEMWBS scores; however, a negative relationship was found at two [*t*_(136)_ = −3.29, *p* < 0.002] and 4 weeks after the psychedelic experience [*t*_(96)_ = −2.61, *p* < 0.020], meaning that having used psychedelic drugs more often in the past had a negative effect on the change in well-being, which also means that having used psychedelics less often, or being completely psychedelic naïve, had a positive effect on the change in well-being after a psychedelic experience.

When further evaluating the estimates of fixed effects, a significant relationship between baseline trait *openness to experience* and baseline WEMWBS scores was found [*t*_(178)_ = 3.16, *p* < 0.003]. Baseline *openness to experience* was also found to predict WEMWBS scores 2 weeks after the psychedelic experience [*t*_(135)_ = 2.19, *p* < 0.040), meaning that those scoring highly on this trait at baseline were more likely to have higher well-being scores at these time-points. Also, the personality trait *emotional stability* [*t*_(178)_ = 2.00, *p* < 0.050], the component “*clear intentions*” [*t*_(178)_ = 2.02, *p* < 0.050] and *visual effects* [*t*_(178)_ = 2.91, *p* < 0.005) were positively related to WEMWBS scores at baseline, but not with well-being measured at later time-points, meaning that individuals scoring high on these variables had higher WEMWBS scores at baseline; however, these variables did not influence changes in well-being after a psychedelic experience. Regarding different intentions to have a psychedelic experience, the component “*spiritual connection*” measured at baseline was positively associated with WEMWBS scores at baseline [*t*_(178)_ = 2.68, *p* < 0.009], and was also predictive of WEMWBS scores two [*t*_(132)_ = 2.11, *p* < 0.040] and 4 weeks after the experience [*t*_(99)_ = 2.13, *p* < 0.040], meaning that individuals who intended to have a psychedelic experience to connect with nature, to have a spiritual experience and/or for therapeutic purposes had overall higher well-being scores at each time-point of measurement. Next, the “*setting*” component was positively associated with WEMWBS scores measured at 2 weeks after the psychedelic experience [*t*_(139)_ = 2.11, *p* < 0.040] and a trend was identifiable at 4 weeks after the psychedelic experience [*t*_(97)_ = 1.78, *p* < 0.080], meaning that feeling comfortable in the environment of the psychedelic experience, and with the people who were present during it, had a positive effect on well-being after the experience. Lastly, an overall positive association between the item “*I am a highly experienced psychedelic drug user*” and WEMWBS scores was found for all time-points, meaning that individuals who consider themselves to be highly experienced with psychedelic drugs had higher well-being scores overall. See Table 6 for the coefficients of the variables included in this model.

**Table 6:** Traits, set and setting, intentions, drug dose, and acute psychedelic experience measures predicting well-being at different time-points.

**Parameter**	**B**	**Std. Error**	***t*-value**	***p*-value**
Intercept	50.329	0.553	116.298	0.000
Time = 4	3.222	0.606	5.317	0.000
Time = 5	3.368	0.720	4.675	0.000
Number of lifetime uses of a psychedelic * Time = 0	0.392	0.553	0.709	0.479
Number of lifetime uses of a psychedelic * Time = 4	−2.215	0.673	−3.289	0.001
Number of lifetime uses of a psychedelic * Time = 5	−2.080	0.798	−2.605	0.011
Bias item^a^ * Time = 0	1.013	0.605	1.675	0.096
Bias item^a^ * Time = 4	1.991	0.754	2.640	0.009
Bias item^a^ * Time = 5	2.588	0.871	2.972	0.004
Openness to experience * Time = 0	1.500	0.475	3.156	0.002
Openness to experience * Time = 4	1.273	0.581	2.193	0.030
Openness to experience * Time = 5	0.166	0.766	0.217	0.829
Emotional stability * Time = 0	1.315	0.656	2.003	0.047
Emotional stability * Time = 4	1.392	0.803	1.734	0.085
Emotional stability * Time = 5	0.943	0.972	0.970	0.335
Trait anxiety * Time = 0	−3.864	0.685	−5.638	0.000
Trait anxiety * Time = 4	−1.188	0.837	−1.419	0.158
Trait anxiety * Time = 5	−2.368	1.022	−2.317	0.023
Suggestibility * Time = 0	0.426	0.472	0.902	0.368
Suggestibility * Time = 4	−0.031	0.583	−0.053	0.957
Suggestibility * Time = 5	0.829	0.716	1.158	0.250
Stubbornness * Time = 0	−0.724	0.473	−1.533	0.127
Stubbornness * Time = 4	−0.894	0.566	−1.581	0.116
Stubbornness * Time = 5	−0.760	0.680	−1.118	0.226
Absorption * Time = 0	−0.085	0.528	−0.162	0.872
Absorption * Time = 4	0.765	0.585	1.308	0.193
Absorption * Time = 5	0.242	0.813	0.297	0.767
“Spiritual connection” * Time = 0	1.428	0.532	2.683	0.008
“Spiritual connection” * Time = 4	1.365	0.646	2.113	0.036
“Spiritual connection” * Time = 5	1.872	0.877	2.134	0.035
“Recreation” * Time = 0	0.005	0.498	0.010	0.992
“Recreation” * Time = 4	0.109	0.639	0.170	0.865
“Recreation” * Time = 5	−0.604	0.777	−0.778	0.438
“Emotional” * Time = 0	−0.688	0.498	−1.382	0.169
“Emotional” * Time = 4	0.006	0.613	0.010	0.992
“Emotional” * Time = 5	−1.348	0.777	−1.735	0.086
“Set”* Time = 0	0.359	0.455	0.789	0.431
“Set”* Time = 4	0.897	0.540	1.662	0.099
“Set”* Time = 5	0.403	0.646	0.624	0.534
“Setting” * Time = 0	0.008	0.460	0.018	0.986
“Setting” * Time = 4	1.206	0.572	2.106	0.037
“Setting” * Time = 5	1.216	0.682	1.784	0.077
“Clear intentions” * Time = 0	0.994	0.491	2.024	0.045
“Clear intentions” * Time = 4	0.340	0.560	0.606	0.545
“Clear intentions” * Time = 5	0.860	0.679	1.267	0.208
Drug dose * Time = 0	−0.867	0.501	−1.730	0.085
Drug dose * Time = 4	0.373	0.588	0.635	0.526
Drug dose * Time = 5	−0.642	0.701	−0.915	0.362
Being in a therapeutic environment * Time = 0	−0.435	0.530	−0.821	0.413
Being in a therapeutic environment * Time = 4	−0.393	0.674	−0.583	0.561
Being in a therapeutic environment * Time = 5	−0.281	0.885	−0.317	0.752
Mystical-type experience * Time = 0	−0.258	0.590	−0.437	0.663
Mystical-type experience * Time = 4	−0.102	0.723	−0.142	0.887
Mystical-type experience * Time = 5	0.002	0.833	0.002	0.998
Challenging experience * Time = 0	−0.191	0.473	−0.403	0.687
Challenging experience * Time = 4	−0.829	0.536	−1.547	0.124
Challenging experience * Time = 5	−0.376	0.697	−0.540	0.591
Visual effects * Time = 0	1.689	0.581	2.906	0.004
Visual effects * Time = 4	1.229	0.699	1.757	0.081
Visual effects * Time = 5	1.459	0.904	1.614	0.110

*Dependent variable: WEMWBS score. Effect of each variable on each time-point is given. Time = 0: baseline, Time = 4: two weeks after the psychedelic experience, Time = 5: 4 weeks after the psychedelic experience*.

a *Bias item: “I am a highly experienced psychedelic drug user”*.

### Predicting acute psychedelic experience measures

The variable *drug dose* and baseline trait *absorption*, measured by the *MODTAS* were found to be predictive of all acute psychedelic experience measures. Positive associations were found between *drug dose* and scores on the MEQ [*F*_(181)_ = 16.07, *p* < 0.001], CEQ [*F*_(181)_ = 8.03, *p* < 0.006] and visual effects [*F*_(181)_ = 21.05, *p* < 0.001], meaning that higher drug doses used for a psychedelic experience were associated with higher scores on all measures of the acute psychedelic experience. Positive associations were found between baseline *absorption* scores and the MEQ [F_(181)_ = 21.11, *p* < 0.001], CEQ [*F*_(181)_ = 6.82, *p* < 0.020] and visual effects [*F*_(181)_ = 8.98, *p* < 0.004], meaning that higher levels of trait *absorption* (i.e., being more susceptible to immersion in certain experiences) measured at baseline were associated with higher scores on all acute psychedelic experience measures.

### Predicting mystical-type experiences

Besides a positive effect of baseline trait *absorption* on the MEQ scores, the results showed that having clear intentions and expectations for the psychedelic experience was also positively associated with mystical-type experience scores indicated by the significant effect of the “*clear intentions*” component [*F*_(181)_ = 6.30, *p* < 0.020]. Lastly, unsurprisingly, a trend towards significance was found for the intention “*spiritual connection*” being predictive of MEQ scores [*F*_(181)_ = 3.75, *p* < 0.060].

### Predicting challenging experiences

Scores on the CEQ were negatively predicted by the “*set*” component [*F*_(181)_ = 21.09, *p* < 0.001], meaning that higher scores of “*set*”, e.g., feeling well prepared and ready for the upcoming experience, were associated with less challenging experiences. Also, the “*recreation*” component underlying the different types of intentions showed a negative association with scores on the CEQ [*F*_(181)_ = 7.72, *p* < 0.007], meaning that those who intended to have the experience for “*recreation*” were less likely to have a challenging experience. Lastly, feeling comfortable in the environment and with the people that were present during the experience, as was measured by the “*setting*” component, was associated with lower scores on the CEQ; however, this effect showed only a trend towards significance [*F*_(181)_ = 3.33, *p* < 0.080].

### Predicting visual effects

Besides the *MODTAS* (*absorption*) and *drug dose*, the “*clear intentions*” component was also positively predictive of visual effects scores [*F*_(181)_ = 4.61, *p* < 0.040], meaning that having clear intentions and expectations for the psychedelic experience was associated with experiencing more perceptual effects during the experience itself.

See Table 7 for the coefficients of the variables included in this model. The variance explained by the current model was highest for the MEQ (*R*^2^ = 0.342), followed by the CEQ (*R*^2^ = 0.324) and visual effects (*R*^2^ = 0.211).

**Table 7:** Traits, set and setting, intentions predicting acute psychedelic experience measures.

**Parameter**	**B**	**Std. Error**	***t*-value**	***p*-value**
**DV: MYSTICAL-TYPE EXPERIENCE**				
Intercept	0.039	0.065	0.596	0.552
Number of lifetime uses of a psychedelic	−0.144	0.083	−1.728	0.086
Bias item^a^	0.079	0.092	0.863	0.389
Openness to experience	−0.071	0.072	−0.992	0.323
Emotional stability	0.032	0.100	0.319	0.750
Trait anxiety	−0.126	0.102	−1.229	0.221
Suggestibility	0.017	0.072	0.242	0.809
Stubbornness	0.020	0.072	0.284	0.777
Absorption	0.347	0.076	4.594	0.000
“Spiritual connection”	0.153	0.079	1.936	0.054
“Recreation”	−0.095	0.074	−1.281	0.202
“Emotional”	0.073	0.075	0.967	0.335
“Set”	0.014	0.065	0.209	0.835
“Setting”	−0.021	0.069	−0.307	0.759
“Clear intentions”	0.180	0.072	2.510	0.013
Drug dose	0.285	0.071	4.009	0.000
Being in a therapeutic environment	0.034	0.080	0.426	0.671
**DV: CHALLENGING EXPERIENCE**				
Intercept	0.099	0.071	1.389	0.166
Number of lifetime uses of a psychedelic	−0.100	0.091	−1.101	0.272
Bias item^a^	−0.022	0.100	−0.219	0.827
Openness to experience	−0.009	0.078	−0.110	0.912
Emotional stability	−0.046	0.108	−0.423	0.673
Trait anxiety	0.140	0.112	1.256	0.211
Suggestibility	0.060	0.078	0.766	0.445
Stubbornness	−0.004	0.078	−0.051	0.960
Absorption	0.215	0.082	2.611	0.010
“Spiritual connection”	0.137	0.086	1.589	0.114
“Recreation”	−0.224	0.081	−2.778	0.006
“Emotional”	0.019	0.082	0.235	0.814
“Set”	−0.324	0.070	−4.593	0.000
“Setting”	−0.137	0.075	−1.824	0.070
“Clear intentions”	−0.134	0.078	−1.714	0.088
Drug dose	0.219	0.077	2.834	0.005
Being in a therapeutic environment	0.021	0.088	0.237	0.813
**DV: VISUAL EFFECTS**				
Intercept	−0.014	0.065	−0.222	0.825
Number of lifetime uses of a psychedelic	−0.116	0.083	−1.387	0.167
Bias item^a^	0.022	0.092	0.237	0.813
Openness to experience	−0.063	0.072	−0.870	0.386
Emotional stability	−0.003	0.100	−0.033	0.974
Trait anxiety	−0.156	0.103	−1.521	0.130
Suggestibility	0.011	0.072	0.152	0.879
Stubbornness	0.060	0.072	0.831	0.407
Absorption	0.227	0.076	2.997	0.003
“Spiritual connection”	−0.033	0.079	−0.412	0.681
“Recreation”	−0.044	0.074	−0.596	0.552
“Emotional”	0.038	0.076	0.501	0.617
“Set”	−0.087	0.065	−1.350	0.179
“Setting”	−0.058	0.069	−0.843	0.400
“Clear intentions”	0.155	0.072	2.148	0.033
Drug dose	0.327	0.071	4.588	0.000
Being in a therapeutic environment	−0.010	0.081	−0.119	0.905

*DV, dependent variable*.

a Bias item: “I am a highly experienced psychedelic drug user.”

## Discussion

The present study used an entirely novel approach to address the important question of how best to predict both acute and longer-term responses to psychedelic compounds. Its primary aim was to test for changes in subjective well-being and then test predictive models of such changes. As hypothesized, well-being was found to be increased 2 weeks after a psychedelic experience and remained increased at 4 weeks. This finding is in line with those of several previous studies (Griffiths et al., 2006, 2011, 2016; Carhart-Harris et al., 2012, 2016; Garcia-Romeu et al., 2014; Schmid et al., 2015), some of which observed improvements in well-being after a single administration of a psychedelic that persisted for over one year (Griffiths et al., 2008; Schmid and Liechti, 2017). Comparing the well-being scores found in the current study to normative WEMWBS data showed that the current sample had baseline well-being scores that were slightly lower than the general population.^7^ However, 2 weeks after the psychedelic experience, the mean well-being score became slightly higher than that for the general population. At 4 weeks after the experience, the mean well-being score was still higher compared to the normative data, although this difference was not significant. Thus, the principle that psychedelics generally enhance psychological well-being, even in already healthy individuals, appears to be supported by the present study's data.

In the current study, we compared individuals showing no change or an increase in well-being to those decreasing in well-being after a psychedelic experience. The only difference found included lower baseline well-being scores in the subsample that showed increases in well-being. This could mean that there was more scope for change after a psychedelic experience in these individuals compared to those who decreased and had a higher baseline level of well-being to begin with. To elaborate on this, we found a modest correlation between the number of lifetime uses of a psychedelic and baseline well-being scores [*r*_(654)_ = 0.21, *p* < 0.001], meaning that those who used psychedelics more often in the past had a higher level of well-being at baseline (see the Supplementary Material for a correlation matrix of the variables of interest described in this study). This positive association between psychedelic use and psychological well-being is supported by a large-scale population study in which suicidality and psychological distress were both lower in psychedelic “users” vs. matched non-users (Hendricks et al., 2015). These findings reinforce the view that psychedelics are an anomaly among drugs of potential misuse, as, with appropriate caveats regarding context of use (Carhart-Harris et al., 2018), use of psychedelics appears to be positively rather than negatively associated with mental health (Hendricks et al., 2015, 2017; Argento et al., 2017; Elsey, 2017; Forstmann and Sagioglou, 2017). Further, a negative correlation between the number of lifetime uses of a psychedelic and change in well-being scores [*r*_(279)_ = –0.23, *p* < 0.001] was found, meaning that those who had more experience with psychedelics in the past showed less improvement in well-being after the psychedelic experience, which is in line with the finding that these individuals had higher well-being scores at baseline.

### Acute psychedelic experience measures as predictors for change in well-being

The present results showed that peak or mystical-type experiences were predictive of positive changes in well-being after a psychedelic experience, challenging experiences were negatively related to well-being at all time-points and visual effects were unrelated to well-being scores after a psychedelic experience. This finding is important as it supports the view that more psychologically complex aspects of the acute psychedelic experience are more influential in determining longer-term outcomes than the arguably more superficial perceptual effects; thus replicating findings from a recent controlled study in patients with depression treated with psilocybin (Roseman et al., 2017). These results could be seen as added justification for the field's present preference for the term “psychedelic” over the historic alternative “hallucinogen”. It has to be emphasized that these two types of experiences, challenging and peak/mystical-type, are not mutually exclusive; both can be experienced to some degree during a psychedelic experience.

Also, the current results are in line with previous findings showing that peak or mystical-type experiences are positively associated with positive longer-term changes after a psychedelic experience (Griffiths et al., 2008, 2011; Garcia-Romeu et al., 2014; Ross et al., 2016; Roseman et al., 2017). As described earlier, the term “mystical-type experience” was popularised by Walter Stace's work on mysticism (Stace, 1960), whereas the term “peak experience” is derived from the work of psychologist Abraham Maslow (Maslow, 1959, 1964), who characterized “peak” as experiencing disorientation in space and time, feelings of being free of inner conflict, feelings of awe, amazement and humility, and a sense of oneness with the universe. Peak experience is effectively synonymous with a mystical-type experience, but could arguably be a more operationally useful term for science (Carhart-Harris and Goodwin, 2017). One might wish to use it in preference to “mystical-type experience” as it may assist the integration of this phenomenon into the scientific mainstream, which may view “mystical” as an inherently problematic term—due to its links with mysticism and associated obscurantism. However, others might argue that it is unnecessarily “political” to use this secular term in preference to a term that has been used widely in the literature, and has a credible research history (Stace, 1960; Hood, 1975; MacLean et al., 2012).

A recent survey study found that the degree of difficulty of a psychedelic experience was positively associated with well-being outcomes (Carbonaro et al., 2016), but the same study also found that longer duration challenging experiences were associated with a subsequent worsening of well-being. The present findings are somewhat more consistent with this latter finding, as we saw a negative effect on well-being when participants rated higher on the challenging experience measure. One way to resolve this apparent paradox may be to construct a new scale that is sensitive to whether or not periods of challenge within a psychedelic experience are overcome—thus, resulting in insight and/or an emotional *breakthrough* or “catharsis.” It may be telling, for example, that the intention to confront difficult emotions was associated with higher scores on the CEQ [*r*_(653)_ = 0.22, *p* < 0.001], but we did not collect any information on whether or not this intention (i.e., to confront difficult emotions) was effectively enacted in the experience itself and whether it was associated with breakthrough and insight. It is possible that a single psychedelic experience can feature, within the same experience, both intense challenge (associated with emotional struggle) and breakthrough—associated with emotional release and subsequent insights and increases in well-being. To date, no measures have been developed to capture this aspect of a psychedelic experience—but we are currently working on rectifying this.

### Traits, set and setting, intentions, and acute psychedelic experience measures predicting well-being changes

This study was to our knowledge the first study combining the prediction of both acute and longer-term responses to psychedelics. Investigating how acute psychedelic experience measures related to longer-term outcomes was the current study's primary aim. However, when building a larger model including different types of variables (i.e., trait variables, set and setting variables, intentions, and acute experience measures) to see how all these different types of variables relate to the change in well-being after a psychedelic experience, it seemed that trait anxiety had the strongest effect on changes in well-being scores. High baseline levels of trait anxiety were associated with low baseline well-being scores. However, at 2 weeks after the psychedelic experience, trait anxiety did not seem to significantly influence well-being scores, meaning that individuals with high levels of trait anxiety at baseline seemed to benefit from a psychedelic experience in such way that the negative influence of trait anxiety on well-being scores was absent at 2 weeks after the psychedelic experience. The positive effect of the psychedelic experience was relatively more short-lived for individuals with higher baseline levels of trait anxiety, as demonstrated by the fact that baseline anxiety also negatively influenced well-being at 4 weeks after the experience (see Table 6). We did not collect any information about if and how individuals dealt with their psychedelic experience afterwards, e.g., if they gained certain insights during their experience and if they had integrated their experience into their daily lives, which is assumed to be a key aspect of the therapeutic process with psychedelics (Pahnke, 1969; Richards, 2015). The only baseline variable influencing change in well-being, besides trait anxiety, was the number of lifetime uses of a psychedelic. This means that those who have used psychedelic compounds less often in the past, or those who were completely psychedelic naïve, showed greater well-being increases after the psychedelic experience.

Perhaps surprisingly, in the more comprehensive model that included both baseline and acute predictors of well-being changes, the acute experience was relatively less predictive of changes in well-being than trait personality factors. Being comfortable in the setting, including being comfortable with people present during the psychedelic experience, was predictive of higher well-being scores measured 2 weeks after the psychedelic experience, which is in line with the assumption that set and setting are important determinants of positive psychedelic experiences (Leary et al., 1963; Hartogsohn, 2016, 2017; Carhart-Harris et al., 2018). The intention to connect with nature and/or to have the experience for spiritual and therapeutic reasons had a positive influence on well-being in general. Having such intentions correlated with the number of lifetime uses of a psychedelic (r_(652)_ = 0.27, *p* < 0.001), indicating that individuals who have used psychedelics more often in the past, were more likely to approach a psychedelic experience with the intention to have a “spiritual connection”, which in turn positively influenced well-being. The current analysis showed that trait, intention and set-and-setting factors might be especially important to consider when predicting longer-term changes after a psychedelic experience and in deciding whether and how to dose with a psychedelic. This is fortuitous in the sense that these are variables that can be assessed and/or introduced ahead of time.

In summary, as with previous studies, we have shown that there is a positive effect of mystical-type or peak experiences on long-term changes after a psychedelic experience (Griffiths et al., 2008; Ross et al., 2016); however, unlike previous studies, by including more and different types of variables into the equation, we have been able to gauge their relative contribution to the overall picture.

### Set, setting, and drug dose as predictors for acute psychedelic experience measures

Importantly, these present results support the long-held (but little tested) assumption that set and setting (Leary et al., 1963; Hartogsohn, 2016, 2017) are key components determining responses to psychedelics (Carhart-Harris et al., 2018). Employing data reduction techniques, we identified three relevant predictive components, namely: “set,” “setting,” and “clear intentions” that were subsequently used to predict the quality of the acute experience. A careful reading of the items that load onto these components can help inform therapeutic strategies for promoting peak or mystical-type and minimizing challenging experiences. For example, it is apparent that making sure an individual feels prepared by promoting clear intentions for having a psychedelic experience is conducive to having a mystical-type experience. *Post-hoc*, we evaluated what intention components correlated with this “clear intentions” component. Both the “spiritual connection” [*r*_(284)_ = 0.19, *p* < 0.001] and “emotional” [*r*_(284)_ = 0.25, *p* < 0.001] intention components showed a positive correlation with “clear intentions.” Whereas “recreation” was negatively associated with ratings of having clear intentions and expectations for the psychedelic experience [*r*_(284)_ = −0.23, *p* < 0.001]. When looking at predictors for having a challenging experience, we saw that being in a good mood, feeling ready and open towards the upcoming experience and low levels of anxiety right before drug intake led to lower ratings of a challenging experience, which is in line with previous research showing that a state of emotional excitability, e.g., being annoyed, angry, nervous, and/or fidgety right before drug intake was predictive of anxious reactions to psilocybin (Studerus et al., 2012). Also, having a “recreation” intention for the psychedelic experience was associated with a lower likelihood of having a challenging experience. This finding may seem somewhat puzzling on first inspection but may relate to the specific content of this complex component, which also includes intentions to have the experience for “social” and “curiosity” based reasons. Thus one might construe these (arguably somewhat vague and ill-defined intentions) as being related to positive, open-minded expectations for the experience that are less to do so with working through psychological strife.

Taken together, the above listed associations may have a degree of obviousness to them, however, they may also be all too easy to neglect in a therapeutic context, e.g., if procedures are rushed and/or care compromised due to external pressures. In the context of controlled research with psychedelics, some examples where the treatment model might be compromised include: (1) because of negative or ill-informed perceptions of the model held by influential figures and/or bodies (Oram, 2012); (2) because of limited resources meaning that e.g., staffing costs cannot be sufficiently subsidised; (3) because of efforts to adhere to a scientifically rigorous or economically cost-effective (but perhaps risky) study design; or (4) because of efforts to rush data collection due to a number of potential pressures.

According to this study's finding, being in a therapeutic environment did not seem to influence the nature of the psychedelic experience in any particular way. This somewhat surprising finding might be interpreted as suggesting that it is not necessarily essential that the environment be carefully designed with therapeutic ends in mind, which would put fewer constraints on the requirements for designing the environment for psychedelic experiences. However, what “being in a therapeutic setting” means to an individual is subjective and variable, and we did not pre-define what we meant by it.

For example, in the present study, individuals in a retreat were grouped together with individuals who reported being in a therapeutic setting. Grouping such scenarios together may be questioned, because retreats do not always adhere to good practice in relation to psychedelic use (Johnson et al., 2008). One should therefore be cautious about inferring too readily from this specific finding. Future work should be done to better define criteria that parse the setting component of the psychedelic experience, and this is something we are presently working on.

Lastly, drug dose was found to be predictive of the quality of the acute psychedelic experience, which is consistent with the findings of previous studies (Hasler et al., 2004; Studerus et al., 2011, 2012; Nour et al., 2016). More specifically, drug dose was predictive of peak or mystical-type (Griffiths et al., 2011) and challenging experiences (Carbonaro et al., 2016), but also of perceptual effects, which is consistent with other studies reporting that all aspects of the acute experience were intensified in a dose-dependent manner (Metzner et al., 1965; Hasler et al., 2004; Studerus et al., 2011).

### Trait-variables as predictors for the acute psychedelic experience measures

Consistent with previous findings (Studerus et al., 2012), the personality trait absorption, which is related to how easily and deeply an individual can be immersed in stimuli and/or experiences, was predictive of all aspects of the acute psychedelic experience measures in this study (i.e., visual effects, mystical-type and challenging experiences). Absorption has been related to genetic variants in the 5-HT2A receptor, with a specific variant relating to greater 5-HT2A receptor signaling being associated with higher absorption scores (Ott et al., 2005). The present finding of the association between absorption and acute psychedelic experiences could be interpreted psychologically, i.e., in terms of a fuller immersion in the psychedelic experience, or neurobiologically, i.e., in terms of potential differences in 5-HT2A receptor functioning facilitating both of these things. These explanations are of course not mutually exclusive. The relevance of personality traits in shaping psychedelic responses has been noted previously (Savage et al., 1966; Studerus et al., 2012) and it is intriguing to think these may have a neurobiological, and more specially, serotonergic underpinning (Frokjaer et al., 2008).

No effects of the other trait variables (i.e., openness to experience, emotional stability, trait anxiety, suggestibility, and stubbornness) on acute psychedelic experience measures were found. This contradicts results of a previous study which found a relationship between the personality trait neuroticism, the inverse of emotional stability, and challenging experiences (Barrett et al., 2017). This different result may be explained by the different study sample. The study of Barrett et al. (2017) recruited a sample consisting of participants that had a challenging experience or “bad trip,” under a psychedelic—indeed, they referred to the survey as “The Bad Trip Survey” (Carbonaro et al., 2016). Given that the Carbonaro et al. study was retrospective in nature, whereas our was prospective, it is possible that participants with higher than average levels of neuroticism were recruited in the former—and one cannot discount the possibility that the “bad trip” itself may have contributed to this potential demographic skew. The prospective nature of the current study eliminates this issue, as it enables us to collect a pre-psychedelic baseline and then track changes from this. Neuroticism scores are known to be higher than average in vulnerable populations (Saklofske et al., 1995; Middeldorp et al., 2006; Distel et al., 2009; Kotov et al., 2010; Thomson, 2016) and published data from our research group shows that, with the right preparations and care, neuroticism can be decreased after treatment with psilocybin for depression (Erritzoe et al., 2018). It might be hasty therefore to include trait neuroticism as an exclusion criterion in future psychedelic studies, particularly if this may be a specific target of the treatment itself. Rather, it may just alert a therapeutic team to the importance of fully harnessing good preparation and other contextual factors to best negotiate any (expected) psychological challenges that may ensue during (and after) the psychedelic session(s).

### Strengths

The present study overcame some of the key challenges inherent in human research with psychedelics by creating a novel study design in which we could sample a large and diverse group of individuals intending on having a psychedelic experience and track them throughout the period from 1 week before until 4 weeks after their experience. To our knowledge, this is a unique design that has never been implemented in psychedelic research before. The present study's prospective design makes it easier to draw inferences about causal relationships, which is a major advantage compared with retrospective survey studies (Carbonaro et al., 2016; Barrett et al., 2017; Johnson et al., 2017).

With its ability to collect large samples and track them in a prospective way, the present approach has considerable potential for gathering more knowledge on the effects of psychedelics (and of course any other intervention or drug), particularly in terms of understanding individual reactions to them, both acutely and longer-term. The current study design allowed us to collect a large amount of data consisting of various types of variables, which could help formulating future research questions. As the development of psychedelics as medicines continues, improved response-prediction tools may prove particularly valuable, for example, in terms of fine-tuning current treatment models, and minimizing potential adverse events associated with psychedelic use. Ultimately, it might be possible to create empirical guidelines that can be revised and improved iteratively, with the ultimate aim of guiding crucial treatment decisions such as whether, when, where and how to dose with a psychedelic.

### Limitations

The present study had several limitations, most of which relate to the web-based observational study design, and associated lack of experimental control. Firstly, participants received links to the relevant surveys on specific moments determined by the anticipated date of their psychedelic experience, which they specified in the sign-up process. However, it was difficult to confirm whether participants complied with instructions to complete the surveys on time. Fortunately, it was possible for participants to adjust the date of their anticipated experience and they were encouraged to contact the study coordinator whenever the date did actually change. However, participants may not have always done so and thus some surveys may have been completed at inappropriate time points. The second survey was a particularly novel aspect of the current design, as it was done just prior to administration of the relevant psychedelic. We were conscious that completing an online survey right before drug intake might present practical challenges in some situations (e.g., in a psychedelic retreat without internet access), therefore it was possible to complete this short survey via pen/pencil and then convert the responses to electronic format at a later, more appropriate, moment. However, we had to take it on trust that participants followed these instructions.

Volunteers signed up for this project if they intended to have a psychedelic experience in the near future. However, such events are not always easily planned and this might have been a reason for potential participants to withdraw from the project prematurely. This may have led to a bias in the sample (see Figure 2) towards those who took their planned experience especially seriously and felt sufficiently motivated and dedicated to see through not just their planned experience but also all of the requested surveys. These conditions may have inadvertently led to a favoring of responses from individuals who prepared well for their experience and were particularly reliable and motivated.

Another limitation that can be acknowledged is sample attrition. Some participants did not complete all surveys, so there might be an inherent selection bias in the reported results. We did not collect any information about why participants decided to drop out and if it had anything to do with the experience or not. It would be an interesting question to investigate if there is a pattern identifiable in baseline information of those participants who did not complete all surveys. This is something we intend to look at in the future.

Further, details about the dose that was used for the psychedelic experience were given after the experience took place. It might therefore be possible, if not likely, that participants' estimate of the dose they had taken was influenced by the intensity of the experienced drug effects, rather than on the actual dose that was administered. This is a common limitation of retrospective data collection. Beyond truly controlled research, in which precision about drug purity and dose can be ensured, one way we might consider overcoming this matter in web-based designs might be to ask for an expected dose a priori, or verification of dose by a sitter, guide or “shaman,” but such measures would likely entail practical challenges and are thus not entirely satisfactory or realistic solutions.

It should also be acknowledged that the validity and concreteness of the concept of “well-being” has been debated in psychology. More work could be done to develop its construct validity, with a view to identifying a more concrete meaning and definition of the term. Future work from our research group will aim to evaluate the impact of psychedelics on different aspects of well-being using different scales that are related to the concept. In this way, we hope to identify different components of well-being that are more or less sensitive to change by psychedelics, and in so doing, reduce some of the term's potential redundancy in this particular context (Carhart-Harris et al., 2017). We also plan to use similar modelling to that used here to predict changes in other phenomena than well-being. Well-being was treated as a useful “catch-all” for psychological health in the present study but it would be interesting to re-analyse these and/or new data to identify anomalous negative adverse reactions—to address for example, factors predictive of increases in schizotypal thinking after a psychedelic experience.

### Implications and future research

The present results highlight a number of variables that may be important to acknowledge when considering how to safely and effectively prepare for and mediate a psychedelic experience. Regarding therapeutic use, our data suggest that efforts to maximize the likelihood of mystical-type experiences are advisable, given that their occurrence is related to larger increases in well-being. Also, creating a comfortable physical and social environment and promoting intentions related to “spiritual connection” for the psychedelic experience seemed to have a direct positive effect on well-being outcomes. Our data also suggest that having clear intentions and positive expectations for the psychedelic experience facilitate the occurrence of mystical-type experiences. Further, feeling ready and prepared for the experience, i.e., having a positive “set,” might have protective effects against having a challenging experience. Also, the data showed that taking these substances with social and/or recreational intentions decreased the likelihood of challenging experiences, perhaps because such individuals were not having the experience in order to work through problematic psychological material. These data also tell us that higher levels of trait absorption and higher drug doses (although there is likely to be an upper limit to the dosage rule) intensify the psychedelic experience, regardless of whether it features mystical or challenging components, which are not mutually exclusive.

Future research should include more and different types of variables to create a broader understanding of the acute and longer-term effects of a psychedelic experience. By including more information, the relative contribution of each type of variables to the complete picture will be clearer, which will be useful for enriching our knowledge about whether, when, where and how to dose with a psychedelic. Further, what a therapeutic environment specifically entails should be investigated more closely. This can be done in a principled way by testing the contribution of different setting variables in relation to certain dependent variables, such as the acute experience and/or longer-term outcomes such as well-being. One example of this is a recent study showing that music, which is a consistent feature in psychedelic therapies, modulates the subjective experience significantly (Kaelen et al., 2015, 2016, 2017b; Lebedev et al., 2016; Preller et al., 2017), and predicts acute mystical-type experience and positive therapy outcomes (Kaelen et al., 2017a).

Also, future research should aim to recruit more heterogeneous study samples and evaluate whether different factors such as superstition, religiosity or culture have different effects on “set” or “pre-state” prior to drug intake, as well as the quality of the subsequent experiences and longer-term outcomes. Further, our efforts to develop an emotional breakthrough questionnaire should help to improve our understanding of the psychology and impact of challenging experiences.

Lastly, to prevent confusion and facilitate a better understanding of psychedelic compounds, future research might strive for consensus in the terminology used to describe peak or mystical-type experiences. Perhaps agreeing, or better still, demonstrating, that the terms are functionally synonymous (if potentially distinct in terms of the surrounding interpretative framework) might be a productive step forwards. We believe that a clearer, mechanistic definition of the peak/mystical-type experience, perhaps with an emphasis on its so-called “unitive” component (Stace, 1960), will help move the phenomenon away from obscurantism and firmly into the scientific arena - which should have a positive naturalizing effect (Carhart-Harris et al., 2017; Roseman et al., 2017).

## Conclusions

In conclusion, this study sought to track individuals intending on having a psychedelic experience to assess pre vs. post-experience changes in well-being and the influence of different predictive factors on this main outcome of interest.

We found that the most potent type of predictors of change in well-being were baseline trait variables. These explained the greatest proportion of the variance in well-being changes, and therefore these will be especially important to consider when deciding to dose with a psychedelic. Of the acute psychedelic experience measures, the occurrence of a mystical-type experience had positive effects on well-being changes, whereas having a challenging experience seemed to have a negative influence on well-being scores in general. Feeling ready for the psychedelic experience and having clear intentions, especially those relating to connecting with nature, spirituality or recreation, were conducive to a peak/mystical-type experience and/or protective against a challenging experience. Scoring high on the trait absorption and higher drug doses intensified the acute experience, increasing the likelihood of having a peak/mystical-type and/or challenging experience. More work is required to examine the association between challenging experiences and longer-term outcomes, as some challenging experiences may have positive aspects to them such as having an emotional breakthrough for example. The present work represents a positive development in terms of reducing uncertainty about how a given individual may respond to a psychedelic but much variance still remains unexplained. Future work, including controlled research, is therefore needed to refine the present model, enabling it to provide more useful information as more data is acquired.

## Author contributions

EH wrote this paper and RC-H edited it with feedback from MK, LR, CT, RD, AH, HK, and RL. All authors read and approved the final manuscript.

### Conflict of interest statement

The authors declare that the research was conducted in the absence of any commercial or financial relationships that could be construed as a potential conflict of interest. The reviewer FB declared a past co-authorship with several of the authors MK, LR, DN, RL, RC-H to the handling Editor.

## Footnotes

^1^www.psychedelicsurvey.com

^2^www.psychedelicsurvey.com

^3^www.reddit.com/r/LSD

^4^www.reddit.com/r/Psychonaut

^5^www.surveygizmo.eu

^6^http://www.mentalhealthpromotion.net/resources/user-guide.pdf

^7^http://www.mentalhealthpromotion.net/resources/user-guide.pdf
