# Supplementary material for: Predicting Responses to Psychedelics: A Prospective Study
Source: Front Pharmacol. 2018 Nov 2;9:897. doi: 10.3389/fphar.2018.00897 (PMC6225734; doi:10.3389/fphar.2018.00897)
Supplement: Supplementary file 1 [file Table_1.docx]

**Supplementary table 1. Correlation matrix**

|  | PPE | BIAS | WB1 | WB_C | TIPI_O | TIPI_E | STAIT | SSS | SOP | MODTAS | INT_S | INT_R | INT_E | SET | SETTING | CL_INT | THERA | DOSE | MEQ | CEQ | VE |
| --- | --- | --- | --- | --- | --- | --- | --- | --- | --- | --- | --- | --- | --- | --- | --- | --- | --- | --- | --- | --- | --- |
| PPE |  | .650  .000  654 | .208  .000  654 | -.226  .000  279 | .145  .000  654 | .120  .002  654 | -.182  .000  654 | -.164  .000  654 | .117  .003  654 | .182  .000  654 | .273  .000  652 | .008  .836  652 | -.093  .017  652 | .132  .027  284 | .125  .035  284 | -.035  .559  284 | -.031  .559  351 | .073  .173  351 | -.008  .889  351 | -.203  .000  351 | -.050  .347  351 |
| BIAS | .650  .000  654 |  | .279  .000  654 | -.146  .014  279 | .171  .000  654 | .203  .000  654 | -.236  .000  654 | -.099  .011  654 | .208  .000  654 | .215  .000  654 | .219  .000  652 | .094  .016  652 | -.093  .018  652 | .185  .002  .284 | .052  .384  284 | -.033  .584  284 | -.038  .482  351 | .125  .019  351 | .103  .055  351 | -.257  .000  351 | .049  .359  351 |
| WB1 | .208  .000  654 | .279  .000  654 |  | -.593  .000  279 | .286  .000  654 | .574  .000  654 | -.697  .000  654 | -.040  .313  654 | .100  .010  654 | .162  .000  654 | .250  .000  652 | .116  .003  652 | -.288  .000  652 | .231  .000  284 | .036  .545  284 | .004  .940  284 | .042  .437  351 | -.068  .202  351 | .135  .012  351 | -.192  .000  351 | .103  .053  351 |
| WB_C | -.226  .000  279 | -.146  .014  279 | -.593  .000  279 |  | -.082  .174  279 | -.263  .000  279 | .426  .000  279 | .014  .818  279 | -.038  .525  279 | .015  .798  279 | -.091  .130  279 | -.036  .549  279 | .186  .002  279 | .005  .952  162 | .143  .070  162 | -.001  .986  162 | .014  .827  253 | .100  .113  253 | .106  .091  253 | -.019  .763  253 | .012  .851  253 |
| TIPI_O | .145  .000  654 | .171  .000  654 | .286  .000  654 | -.082  .174  279 |  | .153  .000  654 | -.174  .000  654 | -.077  .049  654 | .169  .000  654 | .301  .000  654 | .190  .000  652 | -.003  .944  652 | -.011  .770  652 | .117  .050  284 | -.022  .714  284 | .114  .055  284 | .096  .071  351 | .035  .517  351 | .109  .041  351 | -.031  .560  351 | .063  .241  351 |
| TIPI_E | .120  .002  654 | .203  .000  654 | .574  .000  654 | -.263  .000  279 | .153  .000  654 |  | -.707  .000  654 | -.153  .000  654 | .021  .594  654 | .020  .611  654 | .162  .000  652 | .111  .004  652 | -.319  .000  652 | .195  .001  284 | .027  .650  284 | -.089  .136  284 | .042  .432  351 | .021  .701  351 | .069  .196  351 | -.202  .000  351 | .080  .133  351 |
| STAIT | -.182  .000  654 | -.236  .000  654 | -.697  .000  654 | .426  .000  279 | -.174  .000  654 | -.707  .000  654 |  | .131  .001  654 | .003  .937  654 | -.033  .403  654 | -.170  .000  652 | -.116  .003  652 | .363  .000  652 | -.262  .000  284 | -.037  .538  284 | .214  .000  284 | .056  .299  351 | .026  .628  351 | -.039  .469  351 | .215  .000  351 | -.063  .243  351 |
| SSS | -.164  .000  654 | -.099  .011  654 | -.040  .313  654 | .014  .818  279 | -.077  .049  654 | -.153  .000  654 | .131  .001  654 |  | -.175  .000  654 | .144  .000  654 | .003  .938  652 | .174  .000  652 | .091  .020  652 | -.075  .208  284 | -.015  .799  284 | .002  .978  284 | -.027  .611  351 | -.047  .383  351 | .044  .407  351 | .119  .025  351 | -.036  .505  351 |
| SOP | .117  .003  654 | .208  .000  654 | .100  .010  654 | -.038  .525  279 | .169  .000  654 | .021  .594  654 | .003  .937  654 | -.175  .000  654 |  | .293  .000  654 | .123  .002  652 | -.040  .308  652 | .048  .222  652 | .067  .258  284 | .113  .058  284 | .102  .086  284 | .105  .049  351 | .064  .228  351 | .164  .002  351 | -.003  .962  351 | .115  .031  351 |
| MODTAS | .182  .000  654 | .215  .000  654 | .162  .000  654 | .015  .798  279 | .301  .000  654 | .020  .611  654 | -.033  .403  654 | .144  .000  654 | .293  .000  654 |  | .371  .000  652 | .019  .634  652 | .095  .015  652 | .095  .112  284 | .121  .042  284 | .123  .039  284 | .181  .001  351 | .002  .973  351 | .335  .000  351 | .082  .124  351 | .190  .000  351 |
| INT_S | .273  .000  652 | .219  .000  652 | .250  .000  652 | -.091  .130  279 | .190  .000  652 | .162  .000  652 | -.170  .000  652 | .003  .938  652 | .123  .002  652 | .371  .000  652 |  | -.001  .983  652 | .000  .990  652 | .121  .042  284 | .039  .515  284 | .190  .001  284 | .310  .000  351 | .017  .758  351 | .281  .000  351 | .051  .338  351 | .080  .136  351 |
| INT_R | .008  .836  652 | .094  .016  652 | .116  .003  652 | -.036  .549  279 | -.003  .944  652 | .111  .004  652 | -.116  .003  652 | .174  .000  652 | -.040  .308  652 | .019  .634  652 | -.001  .983  652 |  | -.001  .986  652 | .122  .040  284 | .074  .212  284 | -.232  .000  284 | -.353  .000  351 | .082  .125  351 | -.022  .677  351 | -.130  .014  351 | .016  .764  351 |
| INT_E | -.093  .017  652 | -.093  .018  652 | -.288  .000  652 | .186  .002  279 | -.011  .770  652 | -.319  .000  652 | .363  .000  652 | .091  .020  652 | .048  .222  652 | .095  .015  652 | .000  .990  652 | -.001  .986  652 |  | -.093  .118  284 | .021  .720  284 | .252  .000  284 | .282  .000  351 | .043  .425  351 | .053  .325  351 | .084  .114  351 | -.034  .521  351 |
| SET | .132  .027  284 | .185  .002  284 | .231  .000  284 | .005  .952  162 | .117  .050  284 | .195  .001  284 | -.262  .000  284 | -.075  .208  284 | .067  .258  284 | .095  .112  284 | .121  .042  284 | .122  .040  284 | -.093  .118  284 |  | -.052  .357  319 | -.061  .278  319 | -.033  .630  213 | .061  .373  213 | .070  .310  213 | -.332  .000  213 | -.053  .441  213 |
| SETTING | .125  .035  284 | .052  .384  284 | .036  .545  284 | .143  .070  162 | -.022  .714  284 | .027  .650  284 | -.037  .538  284 | -.015  .799  284 | .113  .058  284 | .121  .042  284 | .039  .515  284 | .074  .212  284 | .021  .720  284 | -.052  .357  319 |  | -.002  .973  319 | -.055  .424  213 | .104  .131  213 | .019  .780  213 | -.117  .090  213 | .027  .695  213 |
| CL_INT | -.035  .559  284 | -.033  .584  284 | .004  .940  284 | -.001  .986  162 | .114  .055  284 | -.089  .136  284 | .214  .000  284 | .002  .978  284 | .102  .086  284 | .123  .039  284 | .190  .001  284 | -.232  .000  284 | .252  .000  284 | -.061  .278  319 | -.002  .973  319 |  | .194  .004  213 | -.011  .870  213 | .221  .001  213 | .019  .779  213 | .133  .052  213 |
| THERA | -.031  .559  351 | -.038  .482  351 | .042  .437  351 | .014  .827  253 | .096  .071  351 | .042  .432  351 | .056  .299  351 | -.027  .611  351 | .105  .049  351 | .181  .001  351 | .310  .000  351 | -.353  .000  351 | .282  .000  351 | -.033  .630  213 | -.055  .424  213 | .194  .004  213 |  | .075  .143  379 | .197  .000  379 | .152  .003  379 | .078  .129  379 |
| DOSE | .073  .173  351 | .125  .019  351 | -.068  .202  351 | .100  .113  253 | .035  .517  351 | .021  .701  351 | .026  .628  351 | -.047  .383  351 | .064  .228  351 | .002  .973  351 | .017  .758  351 | .082  .125  351 | .043  .425  351 | .061  .373  213 | .104  .131  213 | -.011  .870  213 | .075  .143  379 |  | .290  .000  379 | .169  .001  379 | .369  .000  379 |
| MEQ | -.008  .889  351 | .103  .055  351 | .135  .012  351 | .106  .091  253 | .109  .041  351 | .069  .196  351 | -.039  .469  351 | .044  .407  351 | .164  .002  351 | .335  .000  351 | .281  .000  351 | -.022  .677  351 | .053  .325  351 | .070  .310  213 | .019  .780  213 | .221  .001  213 | .197  .000  379 | .290  .000  379 |  | .287  .000  379 | .600  .000  379 |
| CEQ | -.203  .000  351 | -.257  .000  351 | -.192  .000  351 | -.019  .763  253 | -.031  .560  351 | -.202  .000  351 | .215  .000  351 | .119  .025  351 | -.003  .962  351 | .082  .124  351 | .051  .338  351 | -.130  .014  351 | .084  .114  351 | -.332  .000  213 | -.117  .090  213 | .019  .779  213 | .152  .003  379 | .169  .001  379 | .287  .000  379 |  | .277  .000  379 |
| VE | -.050  .347  351 | .049  .359  351 | .103  .053  351 | .012  .851  253 | .063  .241  351 | .080  .133  351 | -.063  .243  351 | -.036  .505  351 | .115  .031  351 | .190  .000  351 | .080  .136  351 | .016  .764  351 | -.034  .521  351 | -.053  .441  213 | .027  .695  213 | .133  .052  213 | .078  .129  379 | .369  .000  379 | .600  .000  379 | .277  .000  379 |  |
|  | PPE | BIAS | WB1 | WB_C | TIPI_O | TIPI_E | STAIT | SSS | SOP | MODTAS | INT_S | INT_R | INT_E | SET | SETTING | INT | THERA | DOSE | MEQ | CEQ |  |

Each cell contains the Pearson correlation coefficient (*r*), the *p*-value and the sample size (N), respectively.

PPE: Previous psychedelic experience (i.e. number of lifetime uses of a psychedelic)

BIAS: The item: “*I am a highly experienced psychedelic drug user*”

WB1: WEMWBS scores at baseline

WB_C: Change scores in well-being (i.e. WEMWBS scores two weeks after the psychedelic experience – WEMWBS scores at baseline)

TIPI_O: Openness to experience (subscale from the Ten Item Personality Inventory)

TIPI_E: Emotional stability (subscale from the Ten Item Personality Inventory)

STAIT: Trait anxiety

SSS: Suggestibility

SOP: Stubbornness

MODTAS: Absorption

INT_S: ‘Spiritual connection’ (i.e. component underlying different intentions, found with the principle component analysis)

INT_R: ‘Recreation’ (i.e. component underlying different intentions, found with the principle component analysis)

INT_E: ‘Emotional’ (i.e. component underlying different intentions, found with the principle component analysis)

SET: ‘Set’ (i.e. component underlying set and setting, found with the principle component analysis)

SETTING: ‘Setting’ (i.e. component underlying set and setting, found with the principle component analysis)

CL_INT: ‘Clear intentions’ (i.e. component underlying set and setting, found with the principle component analysis)

THERA: Being in a therapeutic environment

DOSE: Drug dose

MEQ: Total score of the Mystical Experience Questionnaire

CEQ: Total score of the Challenging Experience Questionnaire

VE: Score of Visual effects, constructed from the elementary imagery, complex imagery and audio-visual synaesthesia subscales from the OAV.
